# Once an optimist, always an optimist? Studying cognitive judgment bias in mice

**DOI:** 10.1093/beheco/arac040

**Published:** 2022-06-03

**Authors:** Marko Bračić, Lena Bohn, Viktoria Siewert, Vanessa T von Kortzfleisch, Holger Schielzeth, Sylvia Kaiser, Norbert Sachser, S Helene Richter

**Affiliations:** Department of Behavioural Biology, University of Münster, Münster, Germany; Münster Graduate School of Evolution, University of Münster, Münster, Germany; Department of Behavioural Biology, University of Münster, Münster, Germany; Münster Graduate School of Evolution, University of Münster, Münster, Germany; Department of Behavioural Biology, University of Münster, Münster, Germany; Department of Behavioural Biology, University of Münster, Münster, Germany; Institute of Ecology and Evolution, Friedrich Schiller University Jena, Jena, Germany; Department of Behavioural Biology, University of Münster, Münster, Germany; Münster Graduate School of Evolution, University of Münster, Münster, Germany; Department of Behavioural Biology, University of Münster, Münster, Germany; Münster Graduate School of Evolution, University of Münster, Münster, Germany; Department of Behavioural Biology, University of Münster, Münster, Germany; Münster Graduate School of Evolution, University of Münster, Münster, Germany

**Keywords:** animal personality, anxiety, behavioral repeatability, cognitive bias, decision-making under ambiguity, genotype-environment interaction, judgement bias, spatial learning

## Abstract

Individuals differ in the way they judge ambiguous information: some individuals interpret ambiguous information in a more optimistic, and others in a more pessimistic way. Over the past two decades, such “optimistic” and “pessimistic” cognitive judgment biases (CJBs) have been utilized in animal welfare science as indicators of animals’ emotional states. However, empirical studies on their ecological and evolutionary relevance are still lacking. We, therefore, aimed at transferring the concept of “optimism” and “pessimism” to behavioral ecology and investigated the role of genetic and environmental factors in modulating CJB in mice. In addition, we assessed the temporal stability of individual differences in CJB. We show that the chosen genotypes (C57BL/6J and B6D2F1N) and environments (“scarce” and “complex”) did not have a statistically significant influence on the responses in the CJB test. By contrast, they influenced anxiety-like behavior with C57BL/6J mice and mice from the “complex” environment displaying less anxiety-like behavior than B6D2F1N mice and mice from the “scarce” environment. As the selected genotypes and environments did not explain the existing differences in CJB, future studies might investigate the impact of other genotypes and environmental conditions on CJB, and additionally, elucidate the role of other potential causes like endocrine profiles and epigenetic modifications. Furthermore, we show that individual differences in CJB were repeatable over a period of seven weeks, suggesting that CJB represents a temporally stable trait in laboratory mice. Therefore, we encourage the further study of CJB within an animal personality framework.

## INTRODUCTION

Individuals differ in the way they perceive the world. From human psychological research, it is known that these differences become particularly evident in ambiguous situations in which individuals have to decide between different options. Symbolic for such situations is the often-quoted question: “Is the glass half-full or half-empty?”. Some individuals (i.e., “optimists”) interpret ambiguous information in a more positive, and others (i.e., “pessimists”) in a more negative way; a phenomenon referred to as cognitive judgment bias (CJB) in the scientific literature (Paul et al. 2005; Mendl et al. 2010; Rygula et al. 2013). This CJB framework has been transferred from psychology to animal welfare science in 2004 with the aim of using CJB as an indicator of emotional state in non-human animals (henceforth: animals) (Paul et al. 2005; Bethell 2015; Roelofs et al. 2016; Mendl and Paul 2020). In a seminal study, Harding et al. (2004) developed a paradigm to detect CJB in rats. The authors assessed whether rats behaved as expecting either a positive or a negative outcome in an ambiguous situation. In a first step, rats learned to press a lever for a food reward when a tone of one frequency was played (“go” response), and to refrain from pressing the lever to avoid a punishment when a different-frequency tone was played (“no-go” response). Next, to create an ambiguous situation, intermediate tones were played, and the rats had to decide whether to go and press the lever (“optimistic” decision) or to refrain from pressing it (“pessimistic” decision). Thus, the “optimistic” or “pessimistic” responses in this test served as a measure of the animals’ CJB. Since its introduction, the paradigm has revolutionized animal welfare science and has enabled scientists to distinguish between “optimistic” and “pessimistic” individuals in a variety of different animal species, e.g., in mammals like sheep (Destrez et al. 2014) and pigs (Douglas et al. 2012); in birds like canaries (Lalot et al. 2017) and starlings (Bateson and Matheson 2007); in fish (Laubu et al. 2019); and even in insects (Bateson et al. 2011); for reviews see Raoult et al. (2017), Lagisz et al. (2020), and Neville et al. (2020).

While CJB assessment has become a key technique in animal welfare research, the ecological and evolutionary relevance of CJBs has so far not been properly addressed experimentally (but for theoretical considerations on this topic see Trimmer et al. 2013; Bateson 2016; Crump et al. 2020). Under natural conditions, however, animals are confronted with plenty of different decisions on a daily basis: they need to choose whether or not to retreat during contests with conspecifics, or whether to continue foraging under predation risk. Those decisions are often made in the face of uncertainty and their outcomes can be crucially related to survival and fitness. For example, expecting negative outcomes (i.e., being “pessimistic”) in an environment with high predator density might be advantageous: when hearing a nearby bush rustling, it might be better to flee than to continue foraging. When hearing the same sound in an environment with low predator density, however, being “optimistic” might be beneficial, as an unnecessary flight would be energy costly. From an ecological perspective, “optimistic” and “pessimistic” decision styles may therefore represent adaptive strategies, conferring fitness advantages depending on the ecological context, as suggested by theoretical models (McNamara et al. 2011; Fawcett et al. 2014). Thus, it would be of major interest to explore the ecological relevance of individual differences in CJB. Our study was conceived as groundwork for this endeavor by focusing on the causes underlying optimistic and pessimistic decision-making, as well as on the temporal stability of individual differences in CJB. In the latter approach, we examined the CJB concept from the angle of “animal personality” research, a field that has gained increasing attention in behavioral ecology over the recent years (Dall et al. 2004; Réale et al. 2007).

Regarding the causes of individual differences in CJB, studies in humans suggest the involvement of both environmental and genetic factors (reviewed by Hirsch et al. 2016), with a heritability estimated at 30% (Eley et al. 2008). Also in animals, studies using the CJB paradigm point toward effects of both of these factors: in terms of environmental influences, for example, living together with a social partner (Bučková et al. 2019) or the provision of enrichment (Matheson et al. 2008; Brydges et al. 2011) have induced positive shifts in CJB (i.e., more “optimistic” responses). In turn, the separation from a social partner (Daros et al. 2014) or removal of enrichment (Bateson and Matheson 2007) induced negative shifts (more “pessimistic” responses). This sensitivity to environmental influences underlines that CJB is marked by a high degree of plasticity. Furthermore, there are first indications for genetic effects on CJB in animals, yet, the evidence is much scarcer and inconsistent. For instance, congenitally helpless rats were shown to display a negative CJB compared to non-helpless rats (Enkel and Gholizadeh et al. 2010). Moreover, there are hints that different strains of mice also differ in their CJB (Kloke et al. 2014; Novak et al. 2016; Hintze et al. 2018, but see Krakenberg and von Kortzfleisch et al. 2019). Yet, other studies, e.g., in pigs (Carreras et al. 2016) and red junglefowl (Sorato et al. 2018), did not confirm a link between genetic variability and CJB. Nearly all of these studies, however, concentrate on either genetic *or* environmental factors, thereby not considering more complex interactions between genes and the environment. Empirical approaches combining both aspects are still missing.

Although behavior is marked by plasticity to adaptively cope with changing environmental conditions, it has been recognized that in many animal species, individuals exhibit repeatable behavioral differences independent of features such as sex, age, or size (Dall et al. 2004; Sih et al. 2004; Kaiser and Müller 2021). For example, some individuals are more explorative, some more risk-taking, and some more aggressive than others (Groothuis and Carere 2005; Dammhahn and Almeling 2012). This stability of individual differences in behavior over time and/or across different contexts is widely referred to as “animal personality” (Dall et al. 2004; Réale et al. 2007). In light of such findings, it seems likely that CJB may likewise represent a stable trait. To date, only very few studies addressed this question and found individual differences in CJB to be relatively stable. More specifically, stable inter-individual differences for up to three days have been reported for bottlenose dolphins (Clegg et al. 2017) and house mice (Verjat et al. 2021). Only one study in calves systematically investigated a longer time interval and reports temporal stability of between-individual differences in CJB across 25 days (Lecorps, Weary and Keyserlingk 2018).

Against this background, the aims of the present study were twofold: First, we systematically investigated the influence of the environment and the genetic background on CJB in laboratory mice. Mice of two different strains (C57BL/6J and B6D2F1N) were housed in two different environmental conditions (“scarce” and “complex”) and we assessed their CJB using an automated, touchscreen-based active choice paradigm (Krakenberg and Woigk et al. 2019). Since previous studies indicate that there are differences between mouse strains in CJB (Novak et al. 2016; Hintze et al. 2018) and that environmental enrichment influences CJB positively (Matheson et al. 2008; Brydges et al. 2011), we expected to find similar effects. We selected C57BL/6J and B6D2F1N mice because previous work from our group revealed pronounced behavioral differences between these two strains (von Kortzfleisch et al. 2020). Furthermore, both strains are able to learn our touchscreen-based paradigm and they are visually indistinguishable, which allowed us to perform a blinded study.

Second, we explored whether CJB can be considered a stable trait. Therefore, we measured CJB four times across the course of seven weeks and calculated the repeatability of the mice’s responses as a measure of temporal stability. Based on the literature summarized above, we expected the animals’ responses to be repeatable across this period of several weeks.

To confirm the effects of the selected strains and environments on the animals’ behavioral profiles, we assessed anxiety-like behavior and spatial learning in a battery of standardized tests. As others showed lower levels of anxiety in the C57BL/6J strain (von Kortzfleisch et al. 2020) and mice from an enriched environment (Benaroya-Milshtein et al. 2004; Dar Meshi et al. 2006; Hendershott et al. 2016), we expected such differences also in our study.

## MATERIALS AND METHODS

### Animals and housing conditions

We purchased 36 female C57BL/6J and 36 female B6D2F1N mice from a professional breeder (Charles River Laboratories, Research Models and Services, Germany GmbH, Sulzfeld, Germany) at the age of four weeks (one B6D2F1N mouse died of unknown causes during the first week after arrival). Mice were housed in same-strain groups of three individuals per cage (Makrolon cages type III, 38 × 23 × 15 cm³). To allow for individual identification within cages, all mice received partial ear punches upon arrival. This routine procedure induces only slight and momentary pain (Taitt and Kendall 2019), hence, no analgesic was used. Cages were equipped with wood shavings as bedding material (Allspan, Höveler GmbH & Co. KG, Langenfeld, Germany), a paper towel, a wooden stick, a semi-transparent red plastic shelter (11.1 × 11.1 × 5.5 cm³, Tecniplast Deutschland GmbH, Hohenpeißenberg, Germany) and a semi-transparent red handling tunnel (length: 98.55 mm, diameter: 50.8 mm, ZOONLAB GmbH, Castrop-Rauxel, Germany). Housing rooms were kept at a reversed light/dark cycle of 12:12 h with lights off at 8.00 a.m., a temperature of approximately 23 °C, and relative humidity of about 50%. Water and food (Altromin 1314; Altromin Spezialfutter GmbH & Co. KG, Lage, Germany) were provided ad libitum until the beginning of the experimental phase. During the experimental phase, a restrictive feeding regime was provided, that is animals received food once per day to maintain 90-95% of their ad libitum feeding weights. To prevent individual mice from losing too much weight, the food amount per cage was tailored to the lightest mouse when in doubt. Body weights of mice were monitored daily using a digital scale (resolution: 0.1 g; KERN CM 150-1N pocket balance, KERN & Sohn GmbH, Balingen, Germany). The food restriction schedule aimed to increase the mice’s motivation to work for food rewards, without inducing any known negative impact on their welfare (Feige-Diller et al. 2020). Although the influence of a restricted diet on CJB results is not clear, it is unlikely to be the main predictor (Lecorps et al. 2021). To transfer individuals to the target location (i.e., home cage, transport box, behavioral test apparatuses, scale), we gently guided the mice into the handling tunnel, and carried them within the tunnel, a method suggested to reduce stress compared to tail handling (Gouveia and Hurst 2017).

### Experimental design

**Figure 1:**
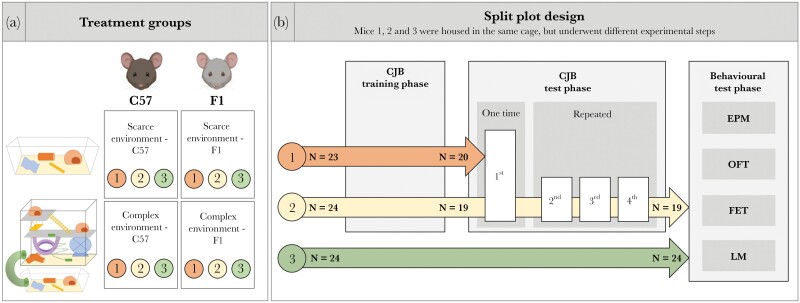
Experimental design. (a) Treatment groups. Mice of two different strains (C57BL/6J and B6D2F1N) were housed under one of two environmental conditions (“scarce” or “complex”). Mice from the “complex” environment had 1 h per day access to the “playgrounds”. The three mice housed in the same cage belonged to the same treatment group but participated in different phases of the experiment. To represent this split plot design, we refer to a subset of mice that had the same experimental procedure with mice “1”, “2”, or “3”. (b) Split plot design. Mice 1 and 2 participated in touchscreen training and the first CJB test. Mice that did not complete touchscreen training were not tested, indicated by the reduced sample sizes (N) after the CJB training phase (for details see section “CJB test”). Mice 1 were relocated and used in another study after the first CJB test. Mice 2 continued with repeated CJB testing and subsequently entered the behavioral test phase together with mice 3. Mice 3 were not exposed to training-related procedures but were otherwise treated as mice 1 and 2. CJB: cognitive judgment bias, EPM: Elevated plus maze, OFT: Open field test, FET: Free exploration test, LM: Labyrinth maze.

We assessed the influence of genetic background and environment on cognitive judgment bias, anxiety-like behavior, and spatial learning by housing mice of two strains in two environmental conditions: a “scarce” environment and a “complex” environment. Half of the mice per strain were assigned to the “scarce” environment. These mice were housed as described above during the whole experimental phase. The other half of the mice were assigned to the “complex” environment. These animals were also housed as described above but had limited access to a super-enriched environment, the “playgrounds”, consisting of varying social and structural elements (for details see section “Complex environmental condition”). Thus, by using a two-by-two full factorial design, four different treatment groups were created (Figure 1a): “scarce” environment C57BL/6J (scarce-C57, *n* = 11), “scarce” environment B6D2F1N (scarce-F1, *n* = 12), “complex” environment C57BL/6J (complex-C57, *n* = 12), and “complex” environment B6D2F1N mice (complex-F1, *n* = 12). To optimize sample sizes per group, we used a design in which mice from each housing cage participated in different sets of the following experimental phases: a touchscreen training phase, first CJB test phase, repeated CJB testing phase, and behavioral test phase (Figure 1b, for details see below).

The experiment lasted for one year (February 2019–March 2020) and was conducted in two independent batches, with the second batch starting three months after the first one. Each batch followed the same experimental procedure and consisted of four different phases: a touchscreen training phase, first CJB test phase, repeated CJB testing phase, and behavioral test phase. The three mice housed in the same cage belonged to the same treatment group but participated in different phases of the experiment (referred to as mouse “1”, mouse “2”, and mouse “3”). This created a split plot design with different sample sizes for each phase (for design details and visualization see Figure 1b): mice “1” were touchscreen trained and tested in the first CJB test phase before they were removed from the cage to participate in another experiment. Mice “2” were touchscreen trained as well. Subsequently, they were repeatedly tested for their CJB and afterwards entered the behavioral test phase. Mice “3” participated only in the behavioral test phase. By having a group of non-trained mice, we were able to control for potential impacts of the touchscreen training on behavioral tests.

The touchscreen training phase started at the age of 10 weeks. Mice participating in this phase underwent daily training sessions to learn the discrimination task required for CJB testing. Once trained mice succeeded in learning the discrimination task with an accuracy of 80% (see Supplementary Table S1 for a detailed training schedule), they entered the CJB testing phase to determine the influence of genotype and environment on CJB. Due to differences in learning speed, mice were differently old when reaching this phase (26 ± 7 weeks). After the first CJB test, one group of mice underwent repeated CJB testing to estimate the repeatability of individual differences in CJB. Subsequently, the repeatedly tested mice, together with the non-trained mice, were tested in a behavioral test battery to investigate the influence of genotype and environment on anxiety-like behavior and spatial learning.

Randomization was performed wherever possible: The allocation of mice to the “scarce” and “complex” environment was done pseudo-randomly. Upon arrival, the mice were distributed to cages so that mice of each strain and environment were represented in each row of the housing rack. To avoid an experimenter bias, we decided to work with C57BL/6J and B6D2F1N strains that have the same fur color and hence are visually indistinguishable. Consequently, experimenters who handled mice did not know which treatment group mice belonged to (blinded study).

### Complex environmental condition

**Figure 2:**
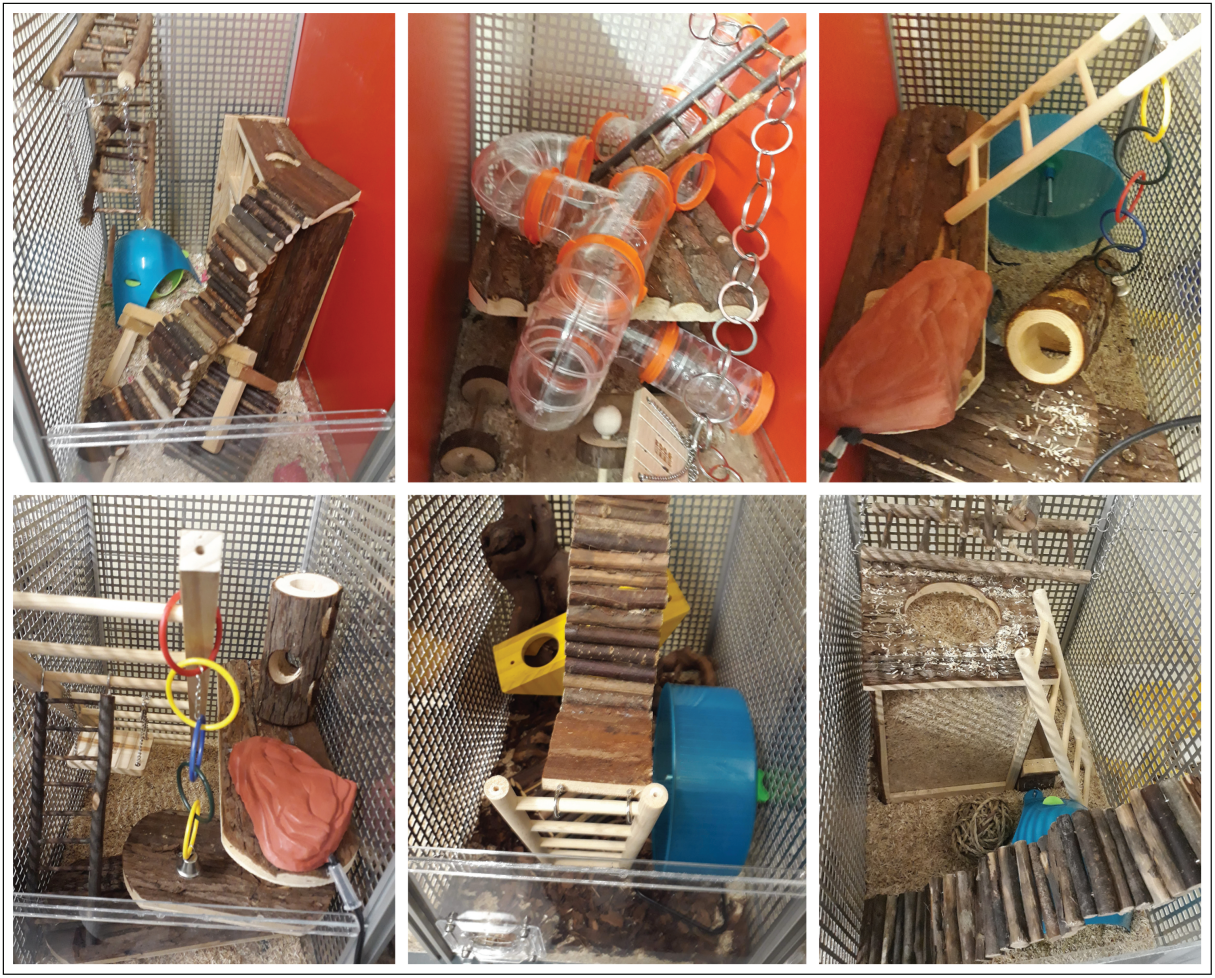
Playgrounds. Example of six differently furnished “playgrounds” used for the “complex” environment. In the social condition, aluminum grid walls were placed between “playgrounds”, while opaque red PVC walls were used to separate the chambers in the non-social condition.

In contrast to the “scarce” environment, the “complex” environment offered mice a highly versatile environment, providing composite structural as well as social enrichment. The system for providing the “complex” environment consisted of six adjacent “playgrounds” (50 × 32 × 52 cm^3^), with a variety of items that allowed mice to express an array of natural behaviors, such as climbing, gnawing, hiding, and digging (Figure 2, Supplementary Video 1). Grid walls between “playgrounds” allowed for tactile, visual, and olfactory contact with individuals other than their cage mates.

Each working day after touchscreen sessions, home cages were connected to one of the “playgrounds” for the duration of 1 h. Cages were taken out of the rack and placed underneath their assigned playground. Each cage had a connector to which a transparent tunnel was attached, connecting the mice’s home cage with the playground. Mice could travel freely between their home cage and their playground. To control for handling effects, cages of the “scarce” environment group were placed on the table next to the “playgrounds” during the same period. After 1 h, all mice received their daily amount of food in the home cage food hopper. When mice left the “playground” to feed (if not, they were gently guided back), the connection tunnel was detached, and cages were returned to the rack. The tunnel connector was closed by a cap (diameter: 6 cm, FPI 4820, Ferplast S.p.A., Castelgomberto, Italy) when not in use.

The limited access to the “playgrounds” was intended to maintain the novelty of this environment during the experimental phase. Maintaining novelty is one of the suggested methods to avoid habituation and loss of interest in enrichment during long-term exposure, by increasing intrinsic motivation to explore novel stimuli (Tarou and Bashaw 2007). To further sustain the novelty of the structural enrichment, each “playground” was furnished differently and mice accessed different “playgrounds” on different days (for different “playground” set-ups see Figure 2). All mice experienced all “playgrounds” and did not encounter the same “playground” more than 2 days in a row (order pseudo-randomized). Additionally, all “playgrounds” were cleaned and furnished with a new set of structural enrichment after six weeks of use.

To sustain the novelty of social enrichment, “playgrounds” were either separated by aluminum grid walls which allowed mice to see and sniff mice from other cages (social condition) or opaque red PVC walls that prevented such contact (non-social condition). Mice did not encounter the same condition for more than three days in a row (order pseudo-randomized; for pictures of “playgrounds” with social and non-social conditions see Figure 2).

### CJB test

#### Apparatus

For the CJB tests and the preceding touchscreen training, we used a commercially available touchscreen system (Bussey-Saksida Mouse Touch Screen Chambers, Model 80614, Campden Instruments Ltd., Loughborough, Uk). The system consisted of four independent chambers. Each chamber was equipped with a tone generator, an overhead illumination, an infrared-sensitive touchscreen at the front, and a reward dispenser with a well for reward collection at the rear end. As a reward, we used servings of diluted sweet condensed milk (Nestlé “Milchmädchen gezuckerte Kondensmilch”, 54.7 g sugar/100 g; diluted 1:4 in tap water). The touchscreen itself was separated into three adjoining windows by a Perspex mask. The central window was used to display cues in the form of white bars (6 × 1 cm²) and the two side windows served as the response windows: mice needed to nose-poke a grey cross (width: 6 cm, height: 6 cm) displayed inside these windows in response to a cue presented in the central window. Data from the touchscreen training and cognitive judgment bias tests were automatically recorded by the ABET II software (version 2.20, Campden Instruments Ltd, Loughborough, Leics, UK).

#### Procedure

During touchscreen training and CJB test phase, mice had one session approximately every 24 h with 1-2 days of a break after five sessions. They were transported individually to the touchscreen system from the housing room using a semi-transparent red transport box. Training sessions ended after max. 30 min or earlier if the scheduled number of trials was reached before that time (for more details on the training schedule, see Krakenberg et al. 2020 and Supplementary Table S1). When the session was finished, mice were carried back to their home cage. After all mice that participated in the touchscreen training were trained, the mice’s weights were recorded and the respective enrichment regime was applied. All touchscreen sessions were conducted after 8.15 a.m. during the dark phase of the reversed daily cycle.

#### Touchscreen paradigm

The paradigm applied here was the same as described previously by Krakenberg et al. (2020) with minor modifications in the discrimination training (Supplementary Table S1). Briefly, mice were trained to discriminate between two reference cues: positive and negative. The positive reference cue was a bar on the lower part of the central window (5 cm below the upper edge) and the negative reference cue was a bar on the upper part of the central window (1 cm below the upper edge). The positions of the positive and negative cue were not counterbalanced in order to minimize variation: pilot data indicated an influence of the association between condition (positive or negative) and reference cue position (top or bottom) on responses to the middle cue, suggesting an asymmetric perception of the cue positions by the mice. In trials with the positive cue, mice received a big reward (12 μL diluted condensed milk) for touching the correct side of the screen or a small reward (4 μL diluted condensed milk) for touching the wrong side. In trials with the negative cue, a bar displayed at the top of the central window, mice received a small reward (4 μL diluted condensed milk) for touching the correct side of the screen or a mild punishment (5-s timeout with lights on) for touching the wrong side. The location of the correct side for the cues was counterbalanced between mice: e.g., one mouse per cage had to touch the right-hand side in response to the positive cue to get a big reward, while the other mouse had to touch the left-hand side in response to the positive cue (two different trial types). The learning criteria and training durations for each step can be found in Supplementary Table S1. Animals that did not finish discrimination training after 90 touchscreen training sessions were excluded from the experiment. From 47 mice trained, 39 successfully finished the discrimination training within 36-90 training days (median 57) and were then tested in the CJB test.

Once mice had learned to discriminate between the positive and negative cues, they proceeded to the CJB test. In the test, mice were presented with ambiguous cues, interspersed between reference cues. As ambiguous cues, we used three bars displayed at three intermediate positions: “near positive” (4 cm below upper edge), “middle” (3 cm below upper edge), and “near negative” (2 cm below upper edge). Using multiple ambiguous cues is recommended to achieve a robust CJB test (Lagisz et al. 2020). In total, the CJB test had 240 reference and 30 ambiguous cue presentations, equally divided into five sessions spread over five days. In each session, each type of ambiguous cue was presented twice and pseudo-randomly interspersed between 48 reference cues: Ambiguous cues appeared only after mice had responded to each reference cue (positive and negative) at least once. They did not appear in direct succession and the preceding cues were counter-balanced so that the same number of positive and negative reference cues was presented directly before each type of ambiguous cue. Responses to ambiguous cues were unrewarded and unpunished. For a demonstration of the paradigm see Supplementary Video 2.

Mice could either react toward the ambiguous cues as if predicting the positive cue outcome by touching the side that was associated with the positive reference cue (“optimistic” choice), or as if predicting the negative cue outcome by touching the side that was associated with the negative reference cue (“pessimistic” choice). Based on their responses during five testing sessions (days), we calculated choice scores for each individual for each ambiguous cue (following Papciak et al. 2013; Rygula et al. 2013; Krakenberg and von Kortzfleisch et al. 2019; Krakenberg and Woigk et al. 2019; Krakenberg et al. 2020):


$$\text{Choice score}=\frac{\text{N choices ("optimistic")-N choices ("pessimistic")}}{\text{N choices }(\text{"optimistic"}+\text{"pessimistic"})}$$

The choice score can take values between −1 and +1, higher values indicating more “optimistic” choices and lower values indicating more “pessimistic” choices. Thus, the choice score serves as a relative behavioral indicator of CJB.

#### Repeated CJB test

After the first CJB test phase, one of two tested mice in each cage was randomly chosen to continue with repeated CJB testing, to estimate the repeatability of individual differences in CJB (*n*_total_ = 19, distributed across experimental conditions as follows: *n*_scarce-C57_ = 3, *n*_complex-C57_ = 6, *n*_scarce-F1_ = 6, *n*_complex-F1_ = 4). The CJB test phase, which itself lasts a week, was repeated three times, with always one non-testing week in-between (following Dingemanse and Wright 2020). Thus, a total of four CJB tests per mouse, resulting in four choice scores per mouse for each cue, was conducted over 7 weeks. During each gap week, mice had two training sessions as reminders to maintain learning accuracy. We scheduled them one day apart and used discrimination training step 6 but without the criterion (see Supplementary Table S1). On the other days during the non-testing week, mice were not trained.

### Battery of behavioral tests

Two weeks after repeated CJB testing, animals (including trained and non-trained mice) were tested in a battery of behavioral tests. The test battery included an elevated plus maze (EPM), an open field test (OFT), and a free exploration test (FET) to assess anxiety-like behavior and exploration (Pellow et al. 1985; Treit and Fundytus 1988; Lister 1990; Griebel et al. 1993). The subsequent labyrinth maze (LM) served to measure spatial learning (Bodden et al. 2019). In total, we measured 13 parameters in the behavioral test battery, which will be specified below.

All tests were performed in a room that met the same conditions as described above for the housing room. Tests were video recorded (Logitech Webcam Pro 9000) and automatically tracked by software (ANY-maze, version 5.33, Stoelting Co., Wood Dale, IL, USA). All setups were cleaned with 70% ethanol between consecutive tests.

Mice were transported into the testing room either in a semi-transparent red transport box (EPM, OFT) or in their home cage covered with a black cloth (FET, LM). When the home cage was used, the test mice’s cage mate(s) were transferred into waiting cages, furnished the same way as their home cage. In the testing room, tested mice had 1 min of waiting time in the transport box to acclimate before being tested. After placing the mice into the start position, the experimenter started the tracking software and left the room (except for the LM, where the experimenter was in the room during the test). All tests were performed during the dark phase between 8.15 a.m. and noon.

#### Elevated plus maze test (EPM)

The apparatus was elevated by 50 cm from the ground and had four arms (30 × 5 cm² each) and a central area (5 × 5 cm²) where the four arms met (Pellow et al. 1985; Lister 1987, 1990). Two opposing arms were enclosed by 20 cm high walls and the other two opposing arms were open. All surfaces of the maze were made of grey PVC. The apparatus was illuminated by an LED lamp producing 25 lux in the central area. For testing, mice were placed in the central area of the apparatus facing the same closed arm. They had 5 min to freely explore the apparatus. The two cage mates (mice “2” and “3”) were tested on the same day. We quantified the relative number of open arm entries and the relative time spent in the open arms as follows:


$$\begin{array}{l} reltive\;\;\;\;open\;arms\;entries=\frac{number\;of\;open\;arms\;entries}{number\;of\;open+closed\;arms\;entries} \end{array}$$

$$\begin{array}{l} relative\;time\;spent\;on\;open\;arms=\frac{time\;spent\;on\;open\;arms}{time\;spent\;on\;open+closed\;arms} \end{array}$$

This allowed us to account for differences in the animals’ overall activity as well as the time they spent in the ambiguous center area. In addition, we assessed the total distance traveled.

#### Open field test (OFT)

The apparatus was a plywood box with a square area (80 × 80 × 42 cm³) painted with white varnish (Archer 1973; Treit and Fundytus 1988). The area 20 cm away from the walls was considered the center zone. The apparatus was illuminated by an LED lamp producing 35 lux in the center. Mice were placed in the front left corner of the apparatus (experimenter’s perspective), facing the corner. Mice had 5 min to freely explore the apparatus. The two cage mates were tested on the same day. We quantified entries into the center zone, time spent in the center zone, and distance traveled.

#### Free exploration test (FET)

The apparatus was a modified version of the open field test which allowed mice to enter the apparatus by choice (Griebel et al. 1993). Light intensity in the center of the arena was set to 35 lux. The apparatus measured 60 × 60 cm² and was framed by 35 cm high walls with an opening in one of them. The opening measured 10 x 15 cm^2^ and was located at the right side of the apparatus (experimenter’s perspective), close to the back-right corner. The mice’s home cage was attached to the opening (during the accommodation time in a transport box) via a transparent tunnel (24 cm × 15 cm × 10 cm). Mice were placed in the home cage and had 15 min to freely explore the apparatus. The two cage mates were tested on consecutive days. We quantified latency to enter the apparatus, number of entries, time spent in the apparatus, and distance traveled.

#### Labyrinth maze (LM)

The apparatus (40 cm × 24 cm) was divided by transparent walls 15 cm in height, forming a labyrinth that offered the mice’s home cage as the goal (Bodden et al. 2019). Light intensity in the center of the arena was set to 12 lux. Mice were placed into the labyrinth and were given a maximum of 5 min to explore the labyrinth and find the exit to their home cage. Once the mice reached their home cage, the home cage was detached from the labyrinth. This test consisted of two trials with a 5-min break in between, in which the mice remained in their home cage and the apparatus was cleaned with 70% ethanol. We quantified latency to reach the home cage, number of mistakes, and distance traveled. A mistake was scored when the mouse either took a wrong passageway or when it took a correct passageway but went back afterward. To evaluate an individual´s learning performance, we calculated the relative difference between the first and second trial for each test measurement by using the following equation:


$$\begin{array}{l} relative\;dif\;ference=\frac{measure\;2st\;trial}{measure\;1nd\;trial} \end{array}$$

Mice improved their performance in the second trial of all three behavioral parameters (Supplementary Table S2). Due to a setup error in the LM, three mice had to be excluded from the LM analysis.

### Data analysis

Data were analyzed using linear mixed-effect models. We assumed a Gaussian distribution and visually checked the distribution of model residuals to confirm reasonable goodness of fit (Schielzeth et al. 2020). When in doubt, we compared model residual histograms of raw and transformed data: if the histograms for models without transformations showed a strong deviation from a normal distribution and the Shapiro–Wilk test (Shapiro and Wilk 1965) was significant, we chose the transformations which produced residual histograms that fit normality assumption best. Where we had multiple measurements per individual, we included individual identifier as random factor to allow for individual differences in intercepts. Similarly, where we had individuals nested in the same cage, we included cage identifier as a random factor to control for pseudoreplication. To calculate *F*-statistics and *P*-values for fixed factors, ANOVA type III tables were produced with sum-contrast coding of fixed factors and the Satterthwaite method for denominator degrees of freedom. Differences were considered statistically significant at *P* ≤ 0.05 (for criticism of using fixed significance level see Wasserstein and Lazar 2016; Wasserstein et al. 2019). For each fixed factor mentioned in the main text, we reported *F* values and *P*-values. As additional information, we also provided model estimates in the form of regression slopes b (which, in the case of two-level factors, are equivalent to treatment differences). For better interpretability of main effect estimates, we centered factors with two levels to 0, coding the two levels as −0.5 (the reference level) and as +0.5 (the contrast level), respectively; thus, slope estimates from the model equal the treatment difference between the factor levels (Schielzeth 2010); similar to sum-contrast coding). We centered fixed factor genotype, environment, and touchscreen training with C57BL/6J strain, “scarce” environment, and “non-trained” as reference levels (three-level factor cue was sum-contrast coded and model estimates not reported). A detailed summary table can be found in Supplementary Table S4.

We conducted an a priori sample-size calculation using the software G*Power (statistics: ANOVA for fixed effects, main effects, and interactions). Data of touchscreen-based CJB tests for mice are very scarce, so it is difficult to properly estimate sample sizes on this basis. Therefore, we based our calculations on the behavioral tests we conducted. Previous data showed that effects of strain (C57BL/6J and B6D2F1N), as well as environmental enrichment on the parameters assessed in tests on anxiety-like behavior are rather large—e.g., effect sizes for large effects between *f* = 0.6–0.8 for strain effects (von Kortzfleisch et al. 2020) and between *f* = 0.5–0.8 for enrichment effects (Bailoo et al. 2018). We here aimed at a power of >80% to detect such large effects (*f* > 0.5), resulting in a total sample size of at least 34 animals. Since sometimes a few animals in CJB tests drop out due to poor learning, we increased the number to 48 animals in total.

### Influence of genotype and environment on CJB

We analyzed influences of genotype, environment, and their interaction on choice scores by using the data from the first CJB test phase and fitting a model with the following factors: cue as fixed within-subject factor (three levels of ambiguous cues: near positive, middle, and near negative); genotype and environment as fixed between-subject factors, including a three-way interaction between cue, genotype, and environment (with all lower-order interactions); and individual and cage as random factors, with individual nested within cage. Before selecting the final model, treatments were assigned at random to the dataset to prevent bias while exploring different models and potential influence of design effects (blind analysis; following MacCoun and Perlmutter 2015). During this blind analysis, we investigated a potential influence of the design effects (modeled as fixed effects): there was no support that either batch (*F* = 0.17, *P* = 0.68), trial type (*F* = 1.76, *P* = 0.20), or training duration (*F* = 0. 57, *P* = 0.46; closely correlated with mouse age at the test) had an effect on the choice score. Thus, these design effects that were not of interest were removed from the final model. In the final analysis, as stated above, we focused only on the ambiguous cues but a model including reference cues was also explored (following Gygax 2014) and it led to the same conclusion.

### Repeatability of CJB

Repeatability is the proportion of total variance in multiple measurements of a trait that is due to differences between individuals. It thus constitutes a useful tool to quantify the stability of individual differences over time (Nakagawa and Schielzeth 2010). The repeatability of CJB was estimated by calculating adjusted repeatabilities (R) of the choice score. The adjusted repeatability removes the fixed effect variance from the estimate (Nakagawa and Schielzeth 2010). We calculated repeatability of choice scores from four CJB tests by fitting a separate model for each ambiguous cue: four repeated CJB tests were modeled as a fixed within-subject continuous variable and individual as random between-subject factor. Additionally, as each individual can respond differently to each cue, we fitted a model with all three ambiguous cues that allows different slopes for each individual across cues. This random slope model resulted in similar repeatability estimates as the above-described models so we report the results of those simpler models, which allowed us to calculate the repeatability for each of the ambiguous cues. The statistical significance of repeatabilities being different from zero was tested by likelihood-ratio tests and uncertainty intervals were estimated by parametric bootstrapping (*n* = 1000, confidence level = 95%).

### Influence of genotype and environment on anxiety-like behavior and spatial learning

We performed a principal component analysis (PCA) based on a total number of 13 behavioral parameters measured in the EPM, OFT, FET, and LM (for direct analysis of the 13 parameters without prior PCA, please see Supplementary data). The Kaiser–Meyer Olkin (KMO) index of sampling adequacy and Bartlett’s test of sphericity (BTS) were used to confirm the appropriateness of this analysis. Absolute parameter loadings of >0.3 were considered relevant for the interpretation of the extracted components.

Three principal components (PC 1–3) explaining more than 72% of the total variance of the data were extracted via visual inspection of the scree plot (Supplementary Figure S1). PC1 explained 31% of the total variance, with negative loadings of parameters from the EPM, OFT, and FET that constitute common indicators of anxiety-like behavior (EPM: relative open arms entries, relative open arms time; OFT entries to the center zone; FET: entries into the arena, distance traveled; for details see Supplementary Figure S1 and Supplementary Table S5). Therefore, we assumed PC1 to reflect “anxiety”. PC2 explained 26% of the total variance, with positive loadings of all three parameters assessed in the LM, and a negative loading of one parameter assessed in the OFT (center time; for details see Supplementary Figure S1 and Supplementary Table S5). Since the absolute loadings of the LM parameters were highest (each > 0.5), we consider PC2 to largely reflect “spatial learning”. PC3 explained 16% of the total variance, with positive loadings of two parameters assessed in the OFT (distance traveled, center entries) and negative loadings of two parameters assessed in the FET (time in arena, distance traveled). The fact that OFT and FET loadings are constructed independently may hint at that PC3 reflects a sub-aspect of “anxiety” which is not explained by PC1. However, to avoid potential misinterpretations about the biological meaning of opposing loadings, we considered that only PC1 and PC2 are of interest in our study and concentrated on these two components in the following.

To investigate whether genotype and environment (interactively) influenced “anxiety” and “spatial learning”, we fitted linear models for PC1 and PC2 with touchscreen training (two levels: trained and non-trained), genotype (two levels: B6D2F1N and C57BL/6J), and environment (two levels: “complex” and “scarce”) as fixed between-subject factors, including a genotype-by-environment interaction, and with cage as a random factor (individuals from the same cage are implicitly nested in the cage factor by sharing the same level). The model for PC2 violated the normality assumption of residuals, so square root transformation was used (after adding a constant equal to the minimal value of PC2).

### Software

Data analysis and plotting were done in R 4.0.0 (R Core Team 2020) with lme4 and lmerTest package for fitting mixed-effect models (Kuznetsova et al. 2017; Bates et al. 2015), obtaining model estimates (b), and producing ANOVA tables, and the rptR package for estimating repeatability (Stoffel et al. 2017). For the PCA, we used the FactoMineR package (Lê et al. 2008), the factoextra package for the scree plot (Kassambara and Mundt 2020), and the psych package for the KMO-criterion and Bartlett calculation (Revelle 2021). Figures were created using the ggplot2 package (Wickham 2016). Sample size calculation was done using G*Power 3.1 (version 3.1.9.7; Faul et al. 2009).

## RESULTS

### CJB was not influenced by the two genotypes or environments

**Figure 3:**
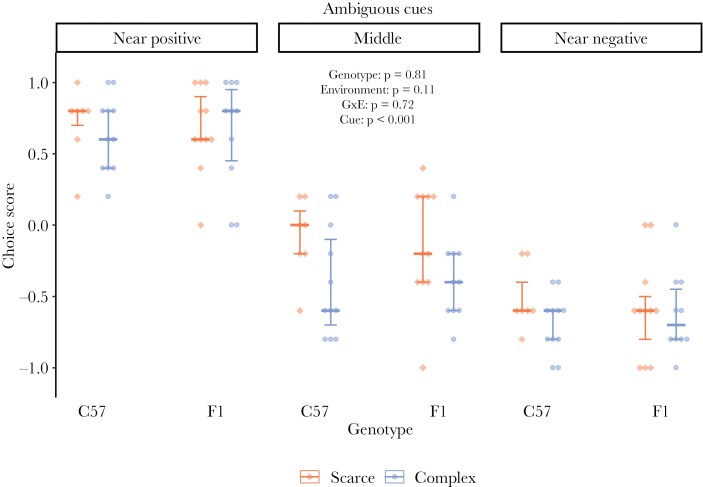
Cognitive judgment bias. Two mouse strains (**C57**BL/6J and B6D2**F1**N) were housed in two environmental conditions: the “scarce” environment (red) and the “complex” environment (blue). Data for each ambiguous cue are presented as medians (horizontal mark) for each treatment group with 25th and 75th percentile as error bars and points for the individual choice score (closer to +1 = more optimistic, closer to -1 = more pessimistic). Statistical analysis was based on the linear mixed-effects model. Number of individuals per treatment: *n*_scarce-C57_ = 7, *n*_complex-C57_ = 11, *n*_scarce-F1_ = 10, *n*_complex-F1_ = 11.

We analyzed the influence of genotype and environment on mice’s reaction toward three ambiguous cues in a touchscreen-based CJB paradigm. There was a statistically significant influence of the three ambiguous cues (*F*_2,76.0_ = 227.77, *P* < 0.001; for a response curve across all cues and a pairwise comparison see Supplementary Figure S2 and Supplementary Table S3). We did not, however, find a statistically significant influence of the two selected genotypes (*b* = −0.02 ± 0.08, *F*_1,19.0_ = 0.06, *P* = 0.81), the two environments (*b* = −0.14 ± 0.08, *F*_1,19.0_ = 2.80, *P* = 0.11), or their interaction (*b* = 0.06 ± 0.17, *F*_1,19.0_ = 0.1, *P* = 0.72) on choice scores (Figure 3). Note that, although the effect of the environment was not statistically significant, mice from the “complex” environment had lower choice scores (*b* = −0.14 ± 0.08). There was also no statistically significant three-way interaction between cue, genotype, and environment (*F*_2,70.0_ = 0.08, *P* = 0.92), or interaction between cue and genotype (*F*_2,70.0_ = 0.01, *P* = 0.98), or between cue and environment (*F*_2,70.0_ = 1.90, *P* = 0.16; see Supplementary Table S4 for ANOVA tables from models reported in this section).

### Individual differences in choice scores were moderately repeatable

**Figure 4:**
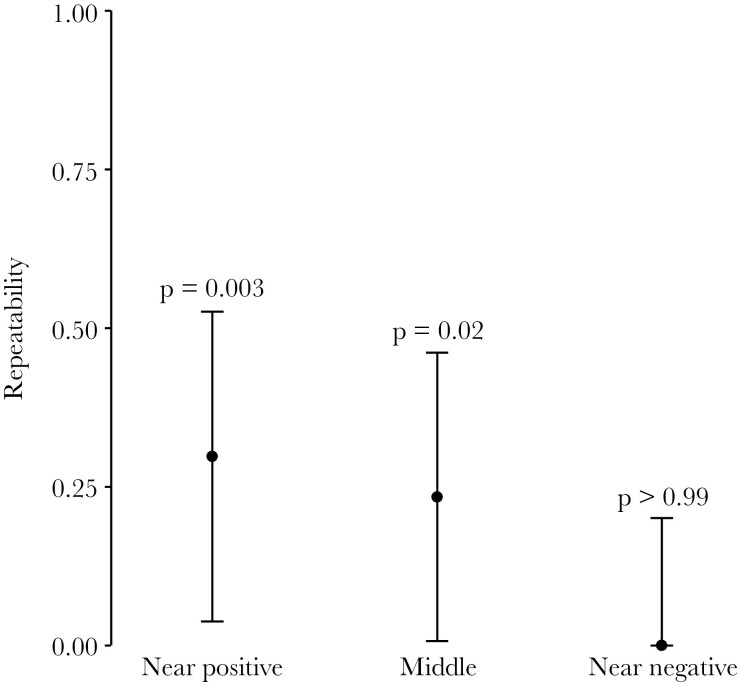
Repeatability of choice scores. Repeatability estimates of mice´s reaction toward three ambiguous cues were based on four CJB tests (*n* = 19). Estimates are represented by dots and their uncertainty with 95% confidence intervals.

To assess the stability of between-individual differences in CJB, we repeated the CJB test four times across the course of 7 weeks and estimated the repeatability of the choice score for each of the three ambiguous cues. Repeatability was estimated at *R* = 0.30 for the “near positive” cue (95% CI [0.04, 0.53], *P* = 0.003) and at *R* = 0.23 for the “middle” cue (95% CI [0.01, 0.46], *P* = 0.02), but at *R* = 0 for the “near negative” cue (95% CI [0.00, 0.20], *P* > 0.99, Figure 4, for visualization of individual choice scores across repeated tests see Supplementary Figure S3).

### Influence of genotype and environment on anxiety-like behavior and spatial learning

Mice were subjected to a battery of four behavioral tests: the elevated plus maze (EPM), the open field test (OFT), the free exploration test (FET), and the labyrinth maze (LM). PCA revealed two biologically meaningful components, PC1, reflecting “anxiety”, and PC2, reflecting “spatial learning”.

#### “Anxiety” (PC1) was influenced by the two genotypes and environments

**Figure 5:**
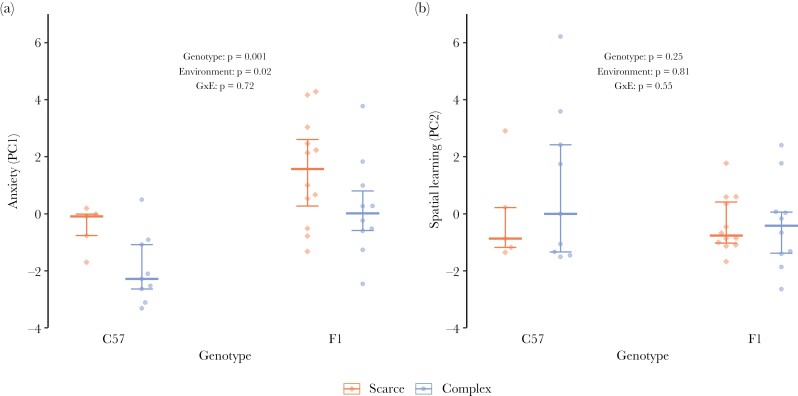
Influence of genotype and environment on “anxiety-like behavior” (PC1) and “spatial learning” (PC2). Two mouse strains (**C57**BL/6J and B6D2**F1**N) were housed in two environmental conditions, “scarce” environment (red) and “complex” environment (blue), and assessed in a behavioral test battery including the EPM, OFT, FET, and LM. Data are represented as medians (horizontal mark) for each treatment group with 25th and 75th percentile as error bars. (a) “Anxiety” (PC1): lower scores indicate lower g performance. Number of individuals: *n* = 36 (per treatment: *n*_scarce-C57_ = 5, *n*_complex-C57_ = 9, *n*_scarce-F1_ = 12, *n*_complex-F1_ = 10).

We found a statistically significant influence of the two genotypes on PC1 (*F*_1,19.6_ = 14.21, *P* = 0.001). Mice from the B6D2F1N strain scored higher on PC1 than mice from the C57BL/6J strain (*b* = 2.09 ± 0.56), indicating that B6D2F1N mice showed higher levels of anxiety-like behavior than did C57BL/6J mice (Figure 5a, Supplementary Table S4).

Moreover, there was a statistically significant influence of the two environments on PC1 (*F*_1,19.7_ = 6.23, *P* = 0.02). Mice from the “complex” environment scored lower on PC1 than mice from the “scarce” environment (*b* = −1.39 ± 0.56), indicating that mice from the “complex” environment showed less anxiety-like behavior than mice from the “scarce” environment (Figure 5a, Supplementary Table S4).

There was no statistically significant effect of touchscreen training on PC1 (*F*_1,19.4_ = 3.29, *p* = 0.09; Supplementary Table S4), but mice that received touchscreen training tended to score higher on PC1 than mice that were not trained (*b* = 0.90 ± 0.49).

#### “Spatial learning” (PC2) was not influenced by the two genotypes or environments

Neither the selected genotypes (*b* = −0.24 ± 0.20, *F*_1,19.1_ = 1.43, *P* = 0.25) nor environments (*b* = −0.05 ± 0.20, *F*_1,19.1_ = 0.06, *P* = 0.81) had a statistically significant influence on PC2 (Figure 5b, Supplementary Table S4), but touchscreen training had (*F*_1,18.3_ = 7.26, *P* = 0.02). Trained mice scored higher on PC2 than non-trained mice (*b* = 0.40 ± 0.15), indicating that non-trained mice learned better than trained mice (Supplementary Table S4).

## DISCUSSION

The assessment of “optimism” and “pessimism” has become an indispensable tool for animal welfare science. Here, we approached the concept from the angle of behavioral ecology and addressed two major aims: First, we studied the roles of genotype, environment, and their interplay on CJB. Second, we explored whether individual differences in CJB are stable over time. To confirm the effects of the selected genotypes and environments on other behaviors, we assessed anxiety-like behavior and spatial learning in a battery of standardized tests. Overall, the two selected genotypes and environments, respectively, did not have a statistically significant influence on the choice score from the CJB test or spatial learning in our laboratory mice, but they did influence anxiety-like behavior. Interestingly, individual differences in the CJB test were moderately repeatable over several weeks, reflecting temporally stable individual differences. Based on these results, we argue that transferring the concept of “optimism” and “pessimism” to behavioral ecology offers great potential for future experimental research: optimistic and pessimistic decision styles could indeed represent different adaptive strategies, entailing fitness consequences depending on an individual’s ecological context (for previous discussions on this topic see also Trimmer et al. 2013; Crump et al. 2020).

### Influence of genotype and environment on CJB

The animals’ reactions toward the five cues in our CJB test produced the expected monotonic response curve, with mice interpreting the three ambiguous cues differently. This typical response curve is in line with the majority of judgment bias tests across species and provides validity that the ambiguous cues are interpreted in relation to the reference cues (Doyle et al. 2010; Lalot et al. 2017; Hintze et al. 2018).

Our study was one of the first to assess the influence of both genetic and environmental factors on CJB in animals within one experiment, thereby allowing us to investigate more complex gene-by-environment interactions. The results showed that neither the selected genotypes, environments, nor their interaction, did significantly influence reactions toward ambiguous cues. This stands in contrast to our expectations since previous studies in humans as well as in animals suggest such influences on CJB (Enkel and Gholizadeh et al. 2010; Hirsch et al. 2016; Lagisz et al. 2020).

Looking at previous studies that investigated the role of genetic factors in modulating CJB in animals, evidence is still inconclusive. While some studies point toward the existence of genetic influences—for example, in rats (Enkel and Spanagel et al. 2010; Richter et al. 2012), mice (Novak et al. 2016; Hintze et al. 2018), or starlings (Bateson et al. 2015)—others did not confirm CJB to have a genetic basis—for example, in pigs (Carreras et al. 2016) or red junglefowl (Sorato et al. 2018). It has thus been suggested that the genetic effect on CJB in animals may be rather low (Sorato et al. 2018). Our results are in line with the latter results, yet, only two genotypes have been studied here and other genotypes might affect CJB differently. The two genotypes were selected because they previously showed pronounced behavioral differences (von Kortzfleisch et al. 2020). For more conclusive evidence about genetic influences, future studies should use a wider range of genotypes or pedigree analyses to estimate the heritability of CJB (following Sorato et al. 2018). Ultimately, artificial selection experiments could aid in ascertaining whether there is a genetic component underlying CJB that is responsive to selection.

Regarding environmental factors influencing CJB in animals, there is more evidence, yet again, results are somewhat inconsistent. While positive effects of enrichment on CJB have been reported in several species of birds and mammals (Matheson et al. 2008; Brydges et al. 2011; Douglas et al. 2012; Richter et al. 2012; Destrez et al. 2014; Löckener et al. 2016; Lalot et al. 2017), a smaller number of studies did not find a beneficial effect of enrichment (reviewed in Lagisz et al. 2020). In line with the latter studies, we also did not detect an influence of versatile structural and social enrichment. There are several possible explanations for our findings, but we consider the following as most likely:

First, in our study, mice had only limited access to the enriched environment (only 1 h per day), which might not have been enough to induce a positive shift of the choice score in the CJB test. In addition, also the animals living in the “scarce” environment were provided with basic enrichment items in their cages (a shelter, a gnawing stick, and nesting material). Thus, the difference between the “scarce” and the “complex” environment might have been smaller than intended, especially when considering that the access to the “playgrounds” was limited. However, mice from the “complex” environment showed lower levels of anxiety-like behavior, indicated by lower scores on PC1, which would suggest that the “playgrounds” still had a positive effect on the mice.

Second, the limited access to the “playgrounds” might have induced a negative contrast effect. More precisely, since the animals were always taken out of the “playgrounds” after 1 h, they might have experienced a withdrawal of enrichment, potentially masking any positive enrichment effects (Latham and Mason 2010).

Third, a recent meta-analysis provided conclusive support that the environment influences CJB (Lagisz et al. 2020), but the effect sizes of environmental manipulation are estimated to be small to moderate (Hedges’ g of 0.2 for the meta-analysis and average Hedges’ *g* of 0.2–0.6 from the individual studies). Thus, stronger manipulations and larger sample sizes might be needed to detect the effects of environmental factors. However, our results could also be explained by a recent theory that differences in CJB will emerge when there is a mismatch between the animals’ expectations and the actual event that follows (for more details, see Raoult et al. 2017 and Eldar et al. 2016).

Based on the current evidence, the story of how one becomes an “optimist” or “pessimist” might thus be more complex than initially assumed; it might be the outcome of a lifelong interplay between (epi-) genetic and numerous, partly stochastic, environmental influences, which cannot be easily disentangled (Lewejohann et al. 2011; Tikhodeyev and Shcherbakova 2019). This assumption should be validated by future studies investigating causes of individual variation in CJB in both sexes and also in wild populations across animal taxa, by using a broader range of environments and genetic backgrounds. A promising approach to identify and quantify the specific drivers of CJB could be to then decompose sources of variation and estimate heritability, methods often used in behavioral ecology and quantitative genetics. Additionally, other potential causes like endocrine profiles and epigenetic modifications could be addressed.

### Temporal stability of individual differences in CJB

Reactions toward the ambiguous cues were repeatable for two out of three ambiguous cues, estimated at *R* = 0.30 for the “near positive” and *R* = 0.23 for the “middle” cue, indicating moderately stable individual differences in CJB over seven weeks. To our knowledge, this is the longest period for which the repeatability of CJB tests has so far been estimated (reviewed by Lecorps et al. 2021). Previous studies covered periods of only 3 days in bottlenose dolphins (corrected *R* = 0.47; Clegg et al. 2017) and mice (*R* = 0.71; Verjat et al. 2021), and up to 25 days in calves (*R*² = 0.41, equivalent to unadjusted repeatability; Lecorps, Weary and Keyserlingk 2018). Rygula et al. (2013) indicated CJB in rats to be stable even across the course of 10 weeks. However, they did not calculate the repeatability of responses to ambiguity and thus did not estimate the magnitude of individual differences. Instead, the authors showed that there was no significant interaction between repeated tests and the assigned CJB category (i.e., “optimistic” or “pessimistic”), indicating temporal stability of individual differences.

Yet, we did not detect significant repeatability of responses to the “near negative” cue (*R* = 0). The reason for this is not clear but might be due to a potentially lower response accuracy toward this cue. Mice showed a lower response accuracy toward the negative than toward the positive reference cue, in our study (see Supplementary Figure S2), as well as in previous studies using the same paradigm (Krakenberg and von Kortzfleisch et al. 2019; Krakenberg and Woigk et al. 2019; Krakenberg et al. 2020). Because the “near negative” cue is visually the most similar to the negative cue, lower accuracy in the negative cue could also lead to reduced accuracy in the “near negative” cue. As reduced accuracy would inflate within-individual variation and hence reduce repeatability (based on the equation for repeatability in Sokal and Rohlf 1995), this paradigm might have underestimated the “true” repeatability for the “near negative” cue.

Overall, our repeatability estimates of *R* = 0.30 and *R* = 0.23 seem to be in a range previously described for other aspects of animal behavior (average *R* = 0.37; Bell et al. 2009). For example, the repeatability for activity and mate preference was estimated at 0.20–0.25, and around 0.5 for aggressive and explorative behavior. Thus, our results align with the notion that CJB does not just reflect a short-lived emotional state directly caused by recent experiences, but also represents a stable trait (Faustino et al. 2015; Mendl and Paul 2020). This finding also embeds individual differences expressed in a CJB test within the concept of animal personality and supports findings linking CJB with other personality traits (Asher et al. 2016; Lecorps and Kappel et al. 2018; Lecorps et al. 2021). We encourage the further study of CJB within the concept of animal personality, as it provides tools to study individual variation within an ecological and evolutionary framework (Dingemanse et al. 2010; Réale et al. 2010).

To further link individual differences in CJB with the concept of animal personality, there are still several open questions to be addressed in future studies: How stable are these differences over even longer periods? Can they be modulated in different life phases? And do they hold across different contexts? To the best of our knowledge, these questions have not been explored yet, although they would provide more information about the trait measured in CJB tests and its potential ecological relevance.

### Influence of genotype and environment on “anxiety”- and “spatial learning”

In our study, both genetic and environmental factors influenced “anxiety” (PC1). C57BL/6J mice scored lower on PC1 than B6D2F1N mice, which indicates lower levels of anxiety-like behavior in C57BL/6J compared to B6D2F1N mice. This is in accordance with a previous study comparing anxiety-like behavior between these two strains (von Kortzfleisch et al. 2020). Furthermore, in comparisons between C57BL/6J and DBA/2 mice, the parental strains of B6D2F1N, C57BL/6J mice expressed lower levels of anxiety-like behavior than DBA/2 mice (Võikar et al. 2005; Bodden et al. 2019, but see Gard et al. 2001).

Furthermore, mice from the complex environment scored lower on PC1, which indicates reduced anxiety-like behavior compared with mice from the “scarce” environment. Again, this is in line with previous studies showing positive effects of environmental enrichment on anxiety (Benaroya-Milshtein et al. 2004; Dar Meshi et al. 2006; Hendershott et al. 2016, but see Kloke et al. 2013; Goes et al. 2015).

Regarding “spatial learning” (PC2), neither genotype nor environment significantly influenced performance in the labyrinth maze. Concerning genotype, to our knowledge, the only study that compared the same two strains in a spatial learning task showed that B6D2F1 mice outperformed both parental strains (Upchurch and Wehner 1989). However, Upchurch and Wehner used different substrains (C57BL/6Ibg and DBA/2Ibg) and a different spatial learning test (the Morris water maze) than we did, so a direct comparison is not possible. Environmental enrichment is known to improve learning performance in mice (Dar Meshi et al. 2006; Loss et al. 2015; Hendershott et al. 2016), so it was surprising not to see a positive effect of environmental enrichment in our study. As discussed for the influence of environmental enrichment on CJB (see section “Influence of genotype and environment on CJB”), one possible reason for the lack of an effect might be the limited access to the enriched environment in our study (for differential effect of exposure time on spatial learning see for example Bennett et al. 2006).

Albeit not in the focus of this study, we controlled for touchscreen training in the statistical models run for PC1 and PC2, since not all mice received training (Figure 1). We found that trained mice scored higher on PC2 (“spatial learning”), indicating a poorer learning performance compared to non-trained mice. Influences of touchscreen training on behavioral and also endocrinological outcome measures have been reported previously; however, the underlying causes are still to be examined (Mallien et al. 2016; Krakenberg et al. 2021). The present findings highlight the importance of accounting for the influence of training procedures, especially when trained and non-trained mice are involved in the same experiment.

## CONCLUSION

We systematically investigated the influence of two genotypes and two environments on cognitive judgment bias, anxiety-like behavior, and spatial learning in laboratory mice. We found that although the selected genotypes and environments influenced some aspects of anxiety-like behaviors, there was no influence of genotype and/or environment on the choice score from the CJB test and spatial learning. Similar discrepancies between CJB and anxiety-like behaviors have already been reported in other studies, indicating that CJB and anxiety may represent distinct systems. Consequently, a “pessimistic” individual might not necessarily be an anxious one.

Furthermore, we provide the first evidence for CJB to be repeatable across several weeks in laboratory mice, indicating that behavioral responses in CJB tests represent a stable trait. On this basis, we suggest that individual differences in CJB reflect not only emotional states but also personality differences in decision-making under ambiguity. However, the proximate causes of these individual differences still need to be elucidated.

Future research should thus aim to identify and quantify the specific drivers of individual differences in CJB. Furthermore, the study of “optimism” and “pessimism” from the perspective of animal personality could provide valuable insights into ecological and evolutionary processes. In particular, empirical studies may answer the question, whether optimists and pessimists do indeed adjust differently to a given environment, and whether they have different social and environmental preferences. Eventually, future studies can contribute to our understanding of the fitness consequences resulting from being an optimist or a pessimist.

## Supplementary data

Supplementary material can be found at http://www.beheco.oxfordjournals.org/

Supplementary Video 1: Playground system for providing complex environment

Supplementary Video 2: Touchscreen-based cognitive judgment bias test

Supplementary Figure S1: Scree plot from PCA on 13 behavioral parameters from the battery of behavioral tests

Supplementary Figure S2: Choice score for each cue in cognitive judgment bias (CJB) test pooled across treatment groups

Supplementary Figure S3: Individual choice scores across repeated cognitive judgment bias (CJB) tests for each cue

Supplementary Table S1: Discrimination training steps

Supplementary Table S2: Spatial learning in the labyrinth maze (LM)

Supplementary Table S3: Summary statistic and pairwise comparison of choice scores for each cue in the first CJB test phase

Supplementary Table S4: Statistical analysis of CJB test and behavioral test battery

Supplementary Table S5: Trait loadings on principal components and component importance from principal component analysis (PCA) of the behavioral test battery

Analysis of behavioral test battery without PCA

arac040_suppl_Supplementary_Video_1

arac040_suppl_Supplementary_Video_2

arac040_suppl_Supplementary_Material

## Data Availability

Analyses reported in this article can be reproduced using the data and scripts provided by Bračić et al. (2022). Author Notes: Marko Bračić and Lena Bohn contributed equally to this work.

## Notes

We thank Eimear Murphy and Luca Melotti for valuable suggestions on the experimental design. We are grateful to Edith Ossendorf, Kimberley Kubski, and Hanna Sicking for the help with the “playgrounds”. Figure 1 used the template “Mouse head (anterior)”, by BioRender.com (2021), retrieved from https://app.biorender.com/biorender-templates.
