# Supplementary material for: Once an optimist, always an optimist? Studying cognitive judgment bias in mice
Source: Behav Ecol. 2022 Jun 3;33(4):775–88. doi: 10.1093/beheco/arac040 (PMC9262167; doi:10.1093/beheco/arac040)
Supplement: arac040_suppl_Supplementary_Material [file arac040_suppl_supplementary_material.pdf]

Supplementary data for manuscript:

Once an Optimist, Always an Optimist? Studying Cognitive Judgment Bias in Mice

Marko Bračić*, Lena Bohn*, Viktoria Siewert[§], Vanessa von Kortzfleisch, Holger Schielzeth, Sylvia Kaiser, Norbert Sachser, S. Helene Richter

*contributed equally to this work, [§]corresponding author

Contents

Supplementary figures 3

Figure S1: Scree plot from PCA on 13 behavioral parameters from the battery of behavioral tests. 3

Figure S2: Choice score for each cue in cognitive judgment bias (CJB) test pooled across treatment groups. 4

Figure S3: Individual choice scores across repeated cognitive judgment bias (CJB) tests for each cue. 5

Supplementary tables 6

Table S1: Discrimination training steps and training durations after Krakenberg et al. (2020) with modifications. 6

Table S2: Spatial learning in the labyrinth maze (LM). 7

Table S3: Summary statistic and pairwise comparison of choice scores for each cue in the first CJB test phase. 8

Table S4: Statistical analysis of CJB test and behavioral test battery. 9

Table S5: Trait loadings on principal components and component importance from principal component analysis (PCA) of the behavioral test battery. 10

Analysis of behavioral test battery without PCA 11

Figure S4: State anxiety. 11

Figure S5: Trait anxiety. 12

Figure S6: Spatial learning. 13

Table S6: Statistical analysis of behavioral test battery without PCA. 14

Supplementary references 18

Supplementary figures

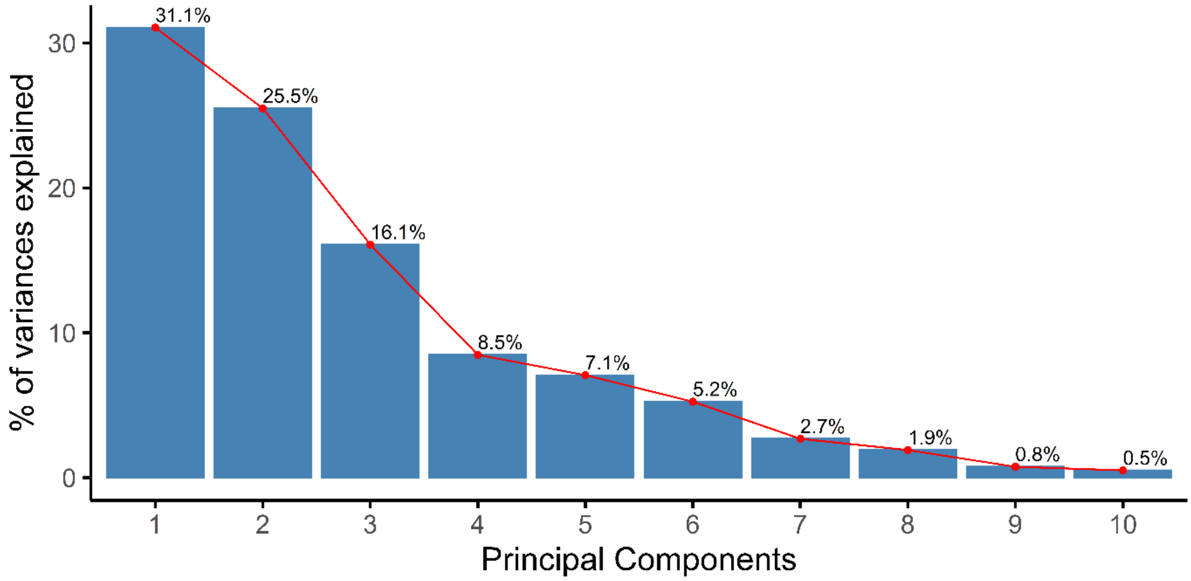

Figure S1: Scree plot from PCA on 13 behavioral parameters from the battery of behavioral tests. Bars and dots show the proportion of variance explained for each of the first 10 principal components. The behavioral test battery included an elevated plus maze, an open field test, a free exploration test, and a labyrinth maze.

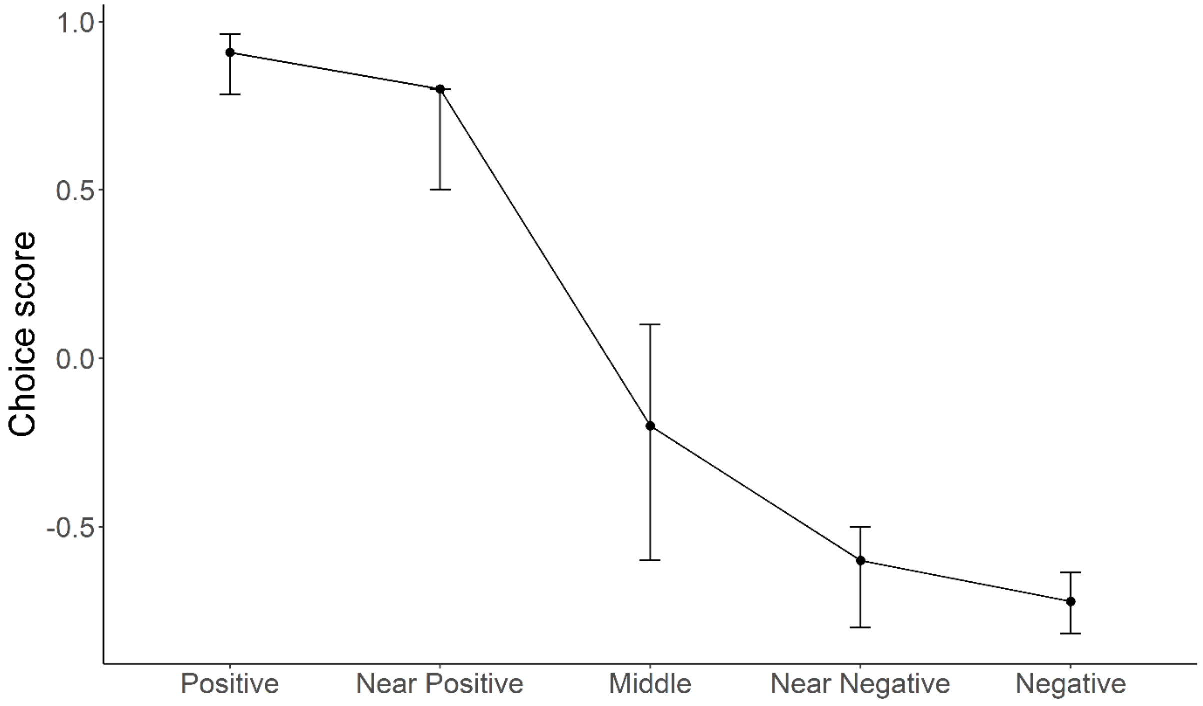

Figure S2: Choice score for each cue in cognitive judgment bias (CJB) test pooled across treatment groups. Displayed are the data from the first CJB test phase, where mice were tested one time in the touchscreen-based CJB paradigm. Data are presented as median (point) for each cue with 25th and 75th percentiles as error bars. Number of individuals: N = 39.

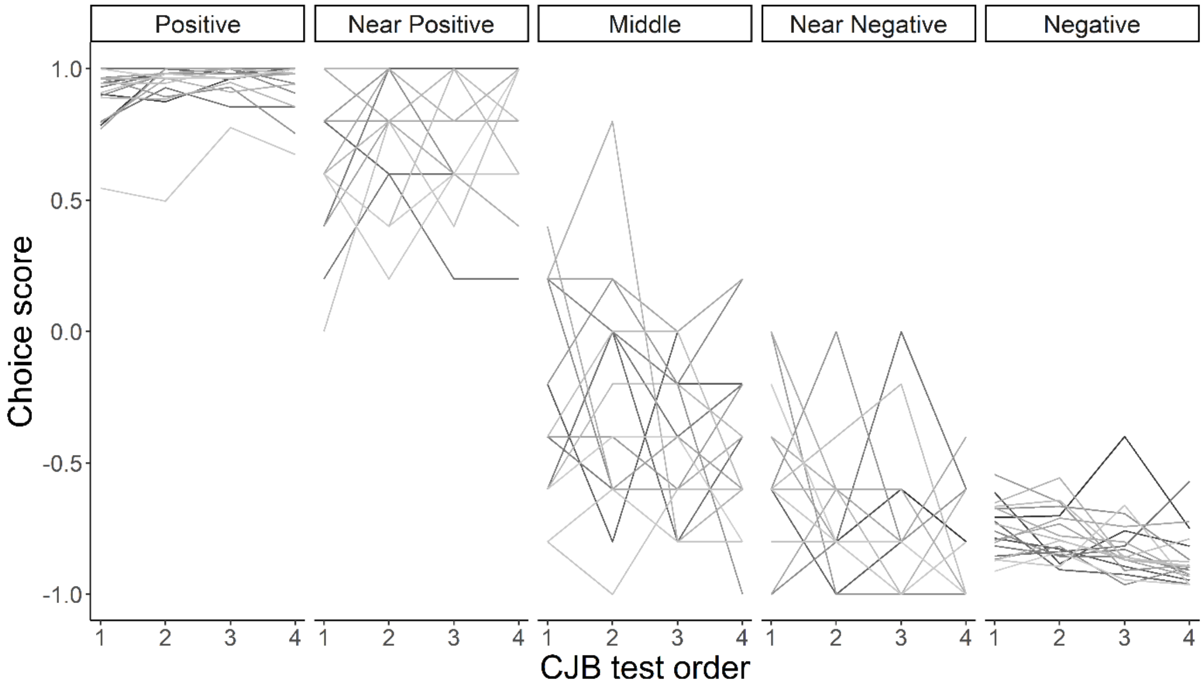

Figure S3: Individual choice scores across repeated cognitive judgment bias (CJB) tests for each cue. Mice were tested four times in the touchscreen-based CJB paradigm. Data are presented as choice scores (lines) for each individual across repeated tests. Number of individuals: N = 19

Supplementary tables

Table S1: Discrimination training steps and training durations after Krakenberg et al. (2020) with modifications. Discrimination training consisted of six steps. All sessions ended after maximally 30 min unless the mouse reached the maximum number of trials before this time. During correction trials (CT) animals were presented with the same cue until touching correctly. Pseudo-probe trials, that is, balanced numbers of positive and negative trials that remained unpunished and/or unrewarded, were included to accustom the mice to the outcome of the probe trials during testing.

Step	Max. number of trials	Learning criterion	Return criterion	Correction trials (CT)	Number of pseudo-probe trials	Number of training days mice needed per step		
						min	max	median
1	50	Minimally 5 days in this step. 50 trials in 20 min on 2 consecutive days	50 trials in 20 min not reached in 25 days → pre-training	-	-	4	31	44
2	20	80% correct responses and ≤7 CTs on two consecutive days	>20 CTs or no CT reduction of 45% daily → Step 1	yes	-	3	49	14
3	50	80% correct responses and ≤13 CTs on two consecutive days	>30 CTs or no CT reduction of 45% daily → Step 1	yes	-	2	13	3
4	50	80% correct responses and ≤8 CTs on two consecutive days	Learning criterion not met on 1 out of 4 days → Step 3	yes (in trials 1-25)	2 (pseudo-randomly distributed across trials 26-50)	2	16	2
5	50	80% correct responses and ≤6 CTs on two consecutive days	Learning criterion not met on 1 out of 4 days → Step 3	yes (in trials 1-15)	4 (randomly distributed across trials 16-50)	2	5	2
6	50	80% correct responses and ≤5 CTs on two consecutive days	Learning criterion not met on 1 out of 4 days → Step 3	yes (in trials 1-5)	6 (randomly distributed across trails 6-50)	3	12	4

Table S2: Spatial learning in the labyrinth maze (LM). To statistically test whether mice improved their performance in the LM from trial 1 to trial 2, we used a Wilcoxon test (paired, N = 40, two-tailed) to compare the measures from trial 1 to the measures from trial 2. Test statistic and p-value were obtained using the wilcoxon.test function in R. Differences were considered statistically significant at $p \leq 0.05$ (bold).

Behavior parameters	Statistic V	p-value
Duration to reach the exit	759	**< 0.001**
Distance travelled	727	**< 0.001**
Number of mistakes	596	**< 0.001**

Table S3: Summary statistic and pairwise comparison of choice scores for each cue in the first CJB test phase. Statistics: Holm-Bonferroni-corrected Wilcoxon signed-rank test (two-tailed). Number of individuals: N = 39. Differences were considered significant at $p \leq 0.05$ (bold).

Cue	Mean	SD	Median	Range (min, max)	Cues compared	p-value
Positive (P)	0.86	0.13	0.91	0.55, 1	P-NP	**< 0.001**
Near Positive (NP)	0.66	0.30	0.80	0, 1	P-M	**< 0.001**
Middle (M)	-0.26	0.37	-0.20	-1, 0.4	P-NN	**< 0.001**
Near Negative (NN)	-0.61	0.28	-0.60	-1, 0	P-N	**< 0.001**
Negative (N)	-0.72	0.13	-0.72	-0.98, -0.36	NP-M	**< 0.001**
					NP-NN	**< 0.001**
					NP-N	**< 0.001**
					M-NN	**< 0.001**
					M-N	**< 0.001**
					NN-N	**0.016**

Table S4: Statistical analysis of CJB test and behavioral test battery. The analysis was based on linear mixed-effects models. To calculate F-statistic and p-values for fixed factors, ANOVA type III tables were produced with sum-contrast coding of fixed factors and the Satterthwaite method for denominator degrees of freedom (using the anova function from lmerTest package in R). Differences were considered statistically significant at $p \leq 0.05$ (bold). Transformation of the response variables was applied where necessary. Full model tests were performed by comparing the full model (including fixed and random factors) with an intercept-only model (including only the random factors).

Fixed factors	Sum Sq	Mean Sq	NumDF	DenDF	F value	p-value	Transformation of the response variable and full model test results
**CJB: choice score (N = 39)**							
Cue	32.242	16.121	2	70.0	227.768	**< 0.001**	None
Genotype	0.004	0.004	1	19.0	0.059	0.811	
Environment	0.198	0.198	1	19.0	2.795	0.111	
GxE interaction	0.009	0.009	1	19.0	0.128	0.724	Full model test:
Cue x GxE interaction	0.012	0.006	2	70.0	0.082	0.921	$Chisq_{11}$ = 180.49
Cue x G interaction	0.002	0.001	2	70.0	0.014	0.986	p **< 0.001**
Cue x E interaction	0.269	0.135	2	70.0	1.903	0.157	
**PC1: anxiety (N = 36)**							
Genotype	30.018	30.018	1	19.6	14.208	**0.001**	None
Environment	13.166	13.166	1	19.7	6.231	**0.022**	
TS training	6.957	6.957	1	19.4	3.293	0.085	Full model test:
GxE interaction	0.271	0.271	1	19.8	0.128	0.724	$Chisq_4$ = 21.35
							p **< 0.001**
**PC2: spatial learning (N = 36)**							
Genotype	0.268	0.268	1	19.1	1.434	0.246	sqrt(PC2+min(PC2))
Environment	0.011	0.011	1	19.1	0.061	0.807	
TS training	1.354	1.354	1	18.3	7.255	**0.015**	Full model test:
GxE interaction	0.069	0.069	1	19.3	0.371	0.550	$Chisq_4$ = 10.04
							p = **0.040**

Table S5: Trait loadings on principal components and component importance from principal component analysis (PCA) of the behavioral test battery. PCA was performed on all 13 measured behavior parameters for the battery of behavioral tests including an elevated plus maze (EPM), an open field test (OFT), a free exploration test (FET), and a labyrinth maze (LM). We used the prcomp function from the stats package in R and extracted values indicating component importance with the summary function. Parameters loading > ± 0.3 (in bold) on PC1 include measures from the test for anxiety-like behavior, parameters loading > ± 0.3 on PC2 (in bold) mainly contain measures from the labyrinth maze.

**Behavioral parameters**	**PC1**	**PC2**	**PC3**	**PC4**	**PC5**	**PC6**	**PC7**	**PC8**	**PC9**	**PC10**	**PC11**	**PC12**	**PC13**
EPM distance travelled	-0.246	-0.093	-0.052	**0.525**	-0.238	**0.698**	-0.302	-0.054	0.106	-0.020	0.041	-0.033	0.017
EPM relative open arm entries	**-0.404**	0.038	0.106	**0.382**	-0.153	**-0.332**	0.037	0.224	**-0.619**	0.181	0.141	0.228	-0.072
EPM relative time spent on open arms	**-0.396**	0.062	0.102	0.291	-0.254	**-0.430**	0.230	-0.074	**0.533**	-0.184	-0.153	-0.272	0.145
OFT distance travelled	-0.172	-0.143	**0.526**	-0.178	0.105	-0.189	**-0.634**	-0.275	0.019	-0.240	0.240	0.037	0.017
OFT entries to the center zone	**-0.306**	-0.248	0.373	-0.208	0.169	0.159	0.023	0.170	0.085	**0.490**	**-0.566**	0.069	0.084
OFT time spent in the center zone	-0.227	**-0.344**	0.287	-0.143	0.161	0.285	**0.549**	0.161	-0.073	-0.361	**0.359**	-0.131	-0.106
FET time spent in the apparatus	-0.279	0.066	**-0.364**	0.064	**0.589**	-0.039	-0.226	**0.405**	0.033	-0.377	-0.144	0.079	0.218
FET entries into the apparatus	**-0.389**	0.066	-0.273	-0.263	-0.046	0.112	0.169	**-0.658**	-0.294	-0.171	-0.221	0.149	0.192
FET distance travelled	**-0.383**	-0.051	**-0.372**	-0.165	0.209	-0.065	-0.081	-0.113	0.210	**0.485**	**0.427**	-0.230	**-0.320**
FET latency to enter the apparatus	0.182	0.124	0.226	**0.506**	**0.629**	-0.010	0.198	**-0.424**	-0.027	0.157	0.003	-0.054	0.013
LM relative difference in duration to reach the exit	-0.146	**0.501**	0.152	-0.056	0.032	0.111	0.041	0.021	0.166	-0.184	-0.172	**0.319**	-0.70
LM relative difference in distance travelled	-0.096	**0.500**	0.175	-0.140	-0.015	0.159	0.121	0.098	0.216	0.200	**0.340**	**0.350**	**0.521**
LM relative difference in number of mistakes	-0.076	**0.507**	0.162	-0.139	-0.000	0.143	-0.091	0.105	-0.317	-0.001	-0.085	**-0.735**	0.056
**Values**													
Standard deviation	2.010	1.821	1.446	1.050	0.959	0.826	0.591	0.498	0.314	0.258	0.186	0.174	0.153
Proportion of Variance	0.311	0.255	0.161	0.085	0.071	0.052	0.027	0.019	0.008	0.005	0.003	0.002	0.002
Cumulative Proportion	0.311	0.566	0.726	0.811	0.882	0.935	0.961	0.981	0.988	0.993	0.996	0.998	1.000

Analysis of behavioral test battery without PCA

To investigate if genotype and environment (interactively) influence the mice's behavior, for each behavioral measurement we fitted a model with touchscreen training (two levels: trained and non-trained), genotype (two levels: B6D2F1N and C57BL/6J), and environment (two levels: complex and scarce) as fixed between-subject factors, including a genotype-by-environment interaction, and with cage as a random factor, with individual nested within cage.

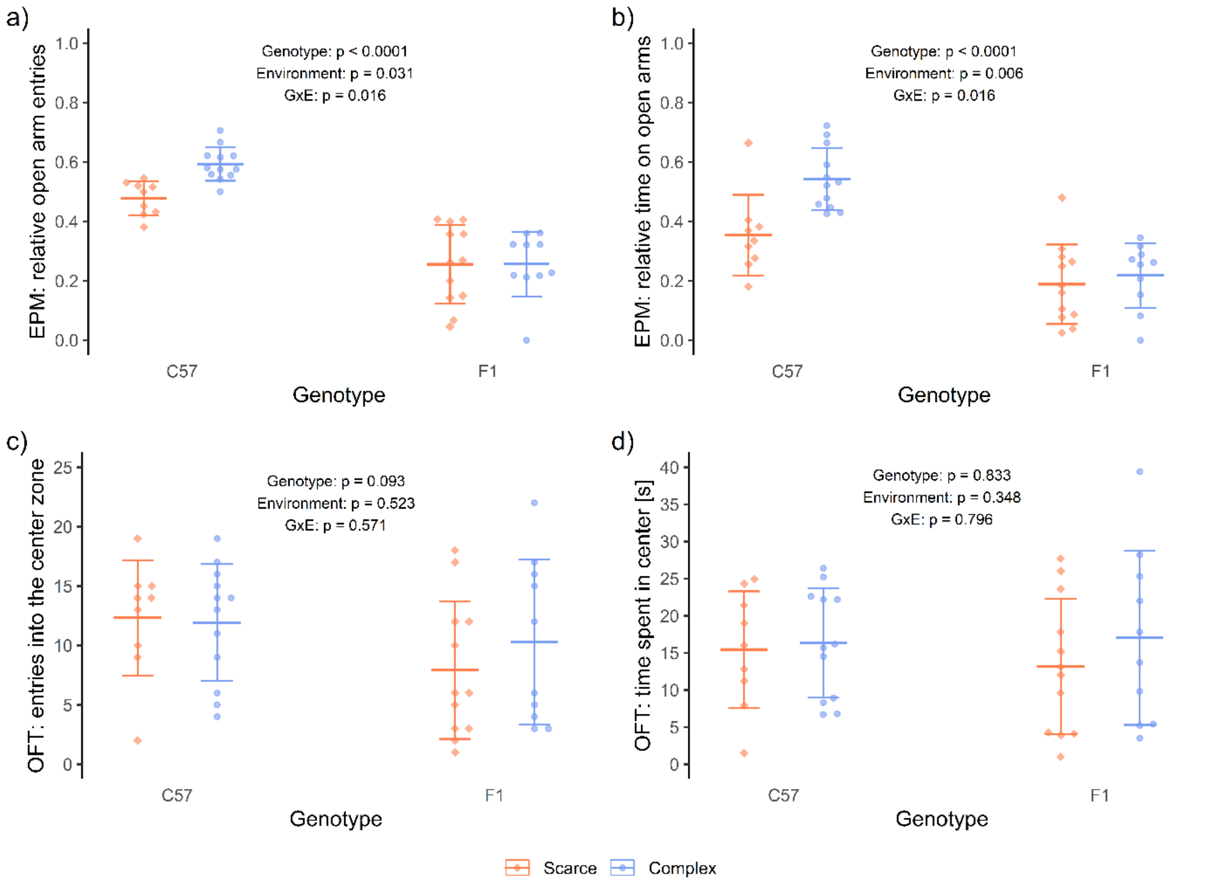

Figure S4: State anxiety. Two mouse strains (C57BL/6J and B6D2F1N) were housed in two environmental conditions: the "scarce environment" (red) and the "complex environment" (blue), and tested in an Elevated plus maze test (EPM) and in an Open field test (OFT) to assess their state anxiety levels. a) number of entries to the open arms relative to the number of closed arm entries on the EPM; b) time mice spent in the open arm relative to the time the mice spent in the closed arm on the EPM; c) number of entries into the center of the OFT; d) time mice spent in the center zone of the OFT. Data are presented as means (horizontal mark) ± SD for each treatment group and points for the individual scores. Statistical analysis was based on the linear mixed-effects models. Number of individuals: Nscarce-C57 = 9, Ncomplex-C57 = 12, Nscarce-F1 = 12, Ncomplex-F1 = 10.

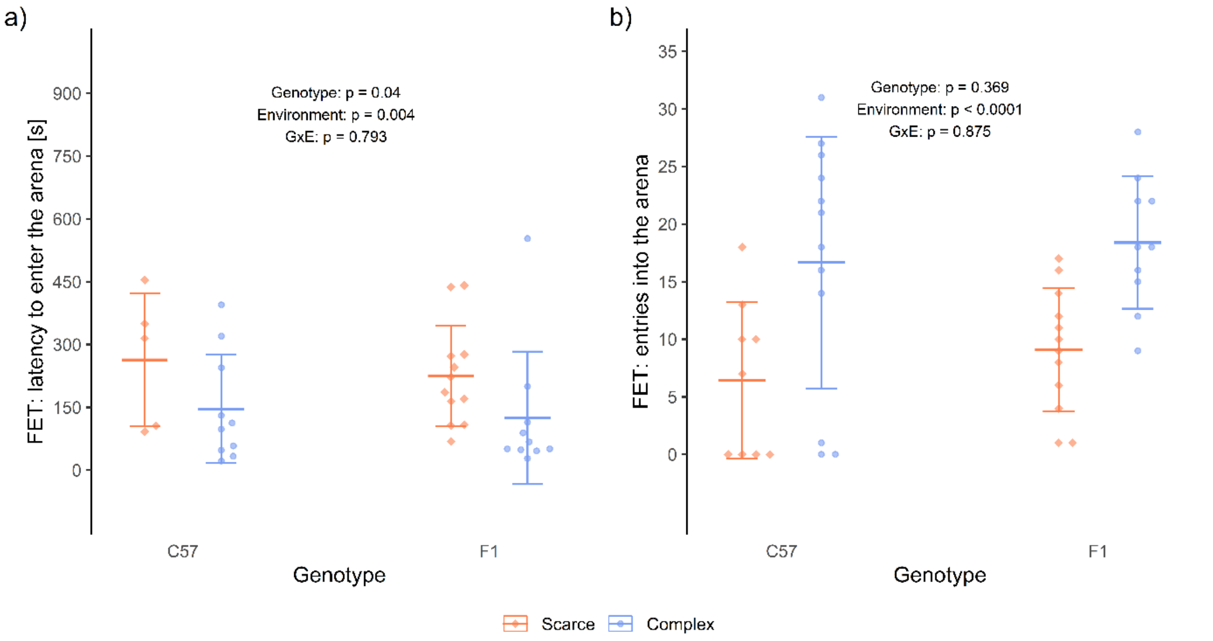

Figure S5: Trait anxiety. Two mouse strains (C57BL/6J and B6D2F1N) were housed in two environmental conditions: the "scarce environment" (red) and the "complex environment" (blue) and tested in a Free exploration test (FET): a) Latency of the mice to first enter the FET arena; b) number of entries into the FET arena from the home cage. Data are presented as means (horizontal mark) ± SD for each treatment group and points for the individual scores. Statistical analysis was based on the linear mixed-effects models. Number of individuals: $N_{scarce\text{-}C57} = 9$, $N_{complex\text{-}C57} = 12$, $N_{scarce\text{-}F1} = 12$, $N_{complex\text{-}F1} = 10$.

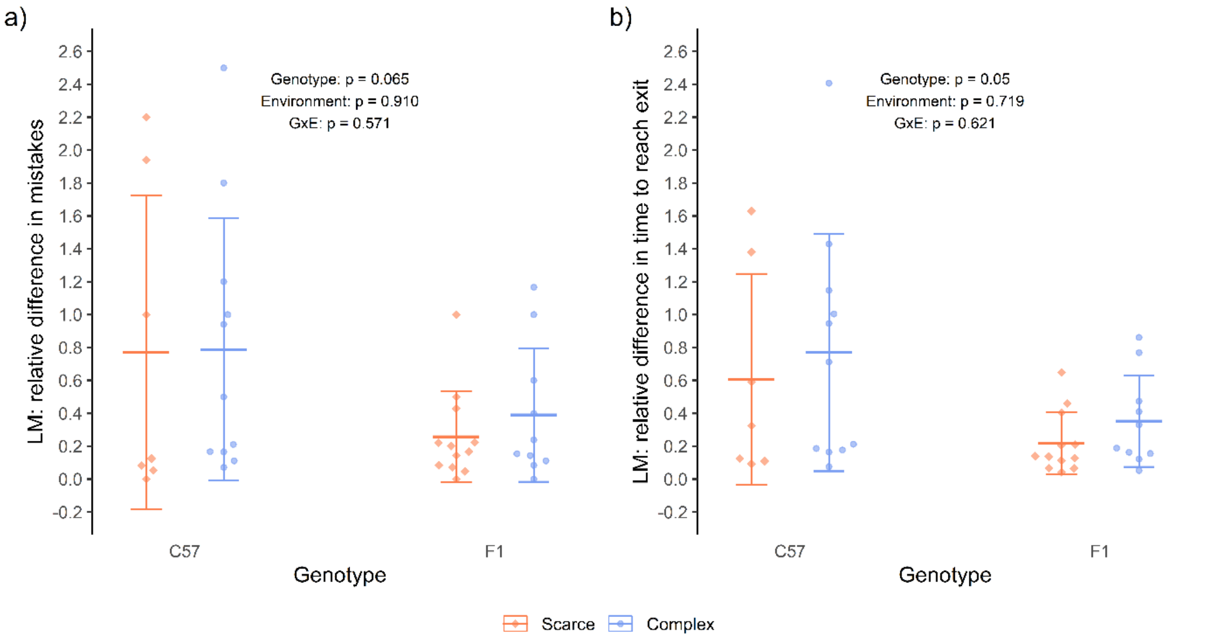

Figure S6: Spatial learning. Two mouse strains (C57BL/6J and B6D2F1N) were housed in two environmental conditions: the "scarce environment" (red) and the "complex environment" (blue) and tested in Labyrinth maze (LM): a) relative difference in mistakes between second and first trial; b) relative difference in time to reach the exit between the second and first trial. Data are presented as means (horizontal mark) ± SD for each treatment group and points for the individual scores. Statistical analysis was based on the linear mixed-effects models. Number of individuals: $N_{scarce\text{-}C57}$ = 7, $N_{complex\text{-}C57}$ = 11, $N_{scarce\text{-}F1}$ = 12, $N_{complex\text{-}F1}$ = 10.

Table S6: Statistical analysis of behavioral test battery without PCA. Analysis was based on the linear mixed-effects models. To calculate F-statistic and p-values for fixed factors, ANOVA type III tables were produced with sum-contrast coding of fixed factors and the Satterthwaite method for denominator degrees of freedom (using anova function from lmerTest package in R). Differences were considered significant at p ≤ 0.05 (bold). Model estimates (b) were obtained using the summary function from of the lmerTest package in R. For better interpretability of main effect estimates, we zero-centered factors with two levels to their mean value: b equals estimated change in the mean value of the factor compared to the reference level when the other factors are at their mean. Fixed between-subject factors were centred for better interpretability of main effect estimates, with C57BL/6J strain, “scarce environment”, and “non-trained” as models’ reference levels. Transformation of the response variables (y) was applied where necessary.

Fixed factors	Estimate (b) ± SE	Sum Sq	Mean Sq	NumDF	DenDF	F-statistic	p-value	Transformation of the response variable (y)
**EPM: entries to open arms (%), N = 43**								
Genotype	-0.276 ± 0.027	0.718	0.718	1	20.1	107.84	**< 0.001**	none
Environment	0.062 ± 0.027	0.036	0.036	1	20.1	5.41	**0.031**	
TS training	-0.095 ± 0.025	0.093	0.093	1	21.8	14.01	**0.001**	
GxE interaction	-0.141 ± 0.054	0.046	0.046	1	20.3	6.87	**0.016**	
**EPM: time spent on open arms (%), N = 43**								
Genotype	-0.244 ± 0.035	0.496	0.496	1	16.1	48.68	**< 0.001**	none
Environment	0.111 ± 0.035	0.103	0.103	1	16.1	10.07	**0.006**	
TS training	-0.113 ± 0.032	0.13	0.13	1	17.5	12.75	**0.002**	
GxE interaction	-0.188 ± 0.070	0.073	0.073	1	16.3	7.15	**0.016**	
**EPM: total distance traveled (m), N = 43**								
Genotype	-8.552 ± 5.486	682.691	682.691	1	20.8	2.43	0.134	$\frac{y^{\lambda}-1}{\lambda}$, λ = 2
Environment	3.239 ± 5.486	97.945	97.945	1	20.8	0.35	0.561	
TS training	-1.751 ± 5.216	31.666	31.666	1	22.5	0.11	0.740	
GxE interaction	-2.974 ± 11.062	20.303	20.303	1	21	0.07	0.791	

Fixed factors	Estimate (b) ± SE	Sum Sq	Mean Sq	NumDF	DenDF	F-statistic	p-value	Transformation of the response variable (y)
**OFT: entries into the centre (#), N = 43**								
Genotype	-2.910 ± 4.736	89.577	89.577	1	38	2.96	0.093	none
Environment	1.090 ± 4.736	12.560	12.560	1	38	0.42	0.523	
TS training	-3. 192 ± 2.196	105.985	105.985	1	38	3.50	0.069	
GxE interaction	1.949 ± 9.500	9.874	9.874	1	38	0.33	0.571	
**OFT: time spent in centre (sec), N = 43**								
Genotype	-0.569 ± 2.680	3.427	3.427	1	38	0.05	0.833	none
Environment	2.547 ± 2.680	68.634	68.634	1	38	0.90	0.348	
TS training	-5.890 ± 2.703	360.766	360.766	1	38	4.75	**0.036**	
GxE interaction	1.404 ± 5.405	5.124	5.124	1	38	0.07	0.796	
**OFT: total distance traveled (m), N = 43**								
Genotype	-6.566 ± 2.838	455.925	455.925	1	38	5.35	**0.026**	none
Environment	0.724 ± 2.838	5.549	5.549	1	38	0.07	0.800	
TS training	-1.563 ± 2.862	25.401	25.401	1	38	0.30	0.588	
GxE interaction	3.528 ± 5.725	32.368	32.368	1	38	0.38	0.541	

Fixed factors	Estimate (b) ± SE	Sum Sq	Mean Sq	NumDF	DenDF	F-statistic	p-value	Transformation of the response variable (y)
**FET: entries into the arena (#), N = 43**								
Genotype	2.168 ± 2.386	49.723	49.723	1	38	0.83	0.369	none
Environment	9.752 ± 2.386	1'005.737	1'005.737	1	38	16.71	**< 0.001**	
TS training	0.535 ± 2.406	2.980	2.980	1	38	0.05	0.825	
GxE interaction	-0.763 ± 4.811	1.513	1.513	1	38	0.03	0.875	
**FET: latency to enter arena (sec), N = 43**								
Genotype	-0.652 ± 0.297	3.979	3.979	1	20.5	4.81	**0.040**	log(y)
Environment	-0.969 ± 0.297	8.790	8.790	1	20.5	10.62	**0.004**	
TS training	-0.068 ± 0.283	0.048	0.048	1	22.2	0.06	0.812	
GxE interaction	0.159 ± 0.599	0.058	0.058	1	20.6	0.07	0.793	
**FET: time spent in the arena (s), N = 43**								
Genotype	39.482 ± 36.050	16'486.37	16'486.366	1	38	1.20	0.280	none
Environment	47.598 ± 36.050	23'961.679	23'961.679	1	38	1.74	0.195	
TS training	32.448 ± 36.355	10'949.928	10'949.928	1	38	0.80	0.378	
GxE interaction	-74.737 ± 72.709	14'522.68	14'522.68	1	38	1.06	0.310	
**FET: total distance traveled (m), N = 43**								
Genotype	3.337 ± 2.671	111.653	111.653	1	19.0	1.56	0.227	none
Environment	7.033 ± 2.671	495.927	495.927	1	19.0	6.93	**0.016**	
TS training	-0.049 ± 2.627	0.025	0.025	1	21.0	0.00	0.985	
GxE interaction	-4.512 ± 5.386	50.191	50.191	1	19.1	0.70	0.413	

Fixed factors	Estimate (b) ± SE	Sum Sq	Mean Sq	NumDF	DenDF	F-statistic	p-value	Transformation of the response variable (y)
**LM: number of mistakes, relative difference (Trial 2 / Trial 1), N = 40**								
Genotype	-0.514 ± 0.261	0.552	0.552	1	18.0	3.87	0.065	none
Environment	-0.030 ± 0.261	0.002	0.002	1	17.9	0.01	0.910	
TS training	0.148 ± 0.127	0.196	0.196	1	16.3	1.37	0.258	
GxE interaction	0.303 ± 0.524	0.048	0.048	1	18.0	0.33	0.571	
**LM: total distance traveled, relative difference (Trial 2 / Trial 1), N = 40**								
Genotype	-0.523 ± 0.473	0.975	0.975	1	16.5	1.22	0.285	log(y)
Environment	0.241 ± 0.473	0.207	0.207	1	16.5	0.26	0.617	
TS training	0.702 ± 0.296	4.516	4.516	1	15.7	5.65	**0.031**	
GxE interaction	0.357 ± 0.095	0.113	0.113	1	16.7	0.14	0.712	
**LM: time to reach exit, relative difference (Trial 2 / Trial 1), N = 40**								
Genotype	-0.268 ± 0.128	0.161	0.161	1	18.2	4.39	0.050	sqrt(y)
Environment	0.047 ± 0.128	0.005	0.005	1	18.2	0.13	0.719	
TS training	0.119 ± 0.065	0.126	0.126	1	16.7	3.45	0.081	
GxE interaction	0.129 ± 0.257	0.009	0.009	1	18.4	0.25	0.621	

Supplementary references

Krakenberg V, Siestrup S, Palme R, Kaiser S, Sachser N, Richter SH. 2020. Effects of different social experiences on emotional state in mice. Sci Rep. 10:1–12.
